# Effects of different exercise intensities or durations on salivary IgA secretion

**DOI:** 10.1007/s00421-024-05467-6

**Published:** 2024-04-18

**Authors:** Takamasa Uchino, Masataka Uchida, Reita Ito, Shumpei Fujie, Keiko Iemitsu, Chihiro Kojima, Mariko Nakamura, Kazuhiro Shimizu, Yuko Tanimura, Yasushi Shinohara, Takeshi Hashimoto, Tadao Isaka, Motoyuki Iemitsu

**Affiliations:** 1 Faculty of Sport and Health Science, Ritsumeikan University, 1-1-1 Nojihigashi, Kusatsu, Shiga 525-8577 Japan; 2 Research Organization of Science and Technology, Ritsumeikan University, Kusatsu, Shiga Japan; 3 Institute of Advanced Research for Sport and Health Science, Ritsumeikan University, Kusatsu, Shiga Japan; 4 Department of Sport Science and Research, Japan Institute of Sports Sciences, Nishigaoka Kita-ku, Tokyo, Japan

**Keywords:** Oral immune function, Open window, Salivary IgA, Exercise intensity, Exercise duration

## Abstract

**Purpose:**

This study aimed to examine changes in salivary immunoglobulin A (s-IgA) secretion at different intensities or durations of acute exercise.

**Methods:**

Twelve healthy untrained young males were included in randomized crossover trials in Experiment 1 (cycling exercise for 30 min at a work rate equivalent to 35%, 55%, and 75% maximal oxygen uptake [${\dot{\text{V}}}$O_2max_]) and Experiment 2 (cycling exercise at 55% ${\dot{\text{V}}}$O_2max_ intensity for 30, 60, and 90 min). Saliva samples were collected at baseline, immediately after, and 60 min after each exercise.

**Results:**

Experiment 1: The percentage change in the s-IgA secretion rate in the 75% ${\dot{\text{V}}}$O_2max_ trial was significantly lower than that in the 55% ${\dot{\text{V}}}$O_2max_ trial immediately after exercise (− 45.7%). The percentage change in the salivary concentration of cortisol, an s-IgA regulating factor, immediately after exercise significantly increased compared to that at baseline in the 75% ${\dot{\text{V}}}$O_2max_ trial (+ 107.6%). A significant negative correlation was observed between the percentage changes in saliva flow rate and salivary cortisol concentration (r = − 0.52, P < 0.01). Experiment 2: The percentage change in the s-IgA secretion rate in the 90-min trial was significantly lower than that in the 30-min trial immediately after exercise (−37.0%). However, the percentage change in salivary cortisol concentration remained the same.

**Conclusion:**

Our findings suggest that a reduction in s-IgA secretion is induced by exercise intensity of greater than or equal to 75% ${\dot{\text{V}}}$O_2max_ for 30 min or exercise duration of greater than or equal to 90 min at 55% ${\dot{\text{V}}}$O_2max_ in healthy untrained young men.

**Supplementary Information:**

The online version contains supplementary material available at 10.1007/s00421-024-05467-6.

## Introduction

Exercise induces significant changes in various biological responses, including circulation, metabolism, nerve activity, and the immune response. Immune function plays an important role in protecting against infections such as bacteria; however, during and/or after exercise, which induces an increase in ventilation, the state of oral immune function is changed. Salivary immunoglobulin A (s-IgA), secreted by oral epithelial cells, is the predominant antibody in mucosal fluid and plays an important role in neutralizing virus and bacteria during oral and suppressing upper respiratory tract infections (Lamm et al.^1995^; Marcotte and Lavoie 1998; Woof and Mestecky 2005). However, long-term and/or high-intensity exercise, such as ultra-marathons, increases the incidence of upper respiratory tract infections by reducing the intraoral s-IgA secretion rate, which is calculated as the product of s-IgA concentration and saliva flow rate (Nieman et al. 2003). The transient depression of s-IgA secretion after a bout of exercise is called the “open window theory”, and this repeated depression may lead to chronic decline in oral immune function (Pedersen and Bruunsgaard 1995). Therefore, it is important to clarify transient changes in s-IgA secretion after exercise to prevent upper respiratory tract infection.

In previous studies on changes in s-IgA secretion after a bout of exercise, the s-IgA secretion rate decreased after high-intensity exercise with 75% maximal oxygen uptake (${\dot{\text{V}}}$O_2max_) (Matsubara et al. 2010; Murase et al. 2016). In contrast, other studies have shown that the s-IgA secretion rate does not decrease even after exercise of the same intensity (Allgrove et al. 2008; Usui et al. 2011). Additionally, moderate-intensity exercise for 120 min reduced the s-IgA secretion rate (Laing et al. 2005); however, the s-IgA secretion rate was not changed by moderate-intensity exercise for 40 min (Mackinnon and Hooper 1994) and 22.3 min (Allgrove et al. 2008). Although it is believed that a reduction in s-IgA secretion may be induced by long-term and/or high-intensity exercise, no consensus has been reached on the effects of exercise intensity and/or duration on s-IgA secretion. As causal factors of these discrepancies in the s-IgA secretion response to exercise, the circadian rhythm of saliva flow rate and s-IgA concentration (Hucklebridge et al. 1998; Dimitriou et al. 2002); water intake during and before/after exercise (Bishop et al. 2000); nutritional status of subjects (Gleeson et al. 2001); and saliva collection methods (Bishop and Gleeson 2009) affect the s-IgA secretion rate. In addition, Bishop and Gleeson (2009) pointed out the importance of evaluating the s-IgA secretion rate among the same subjects (Bishop and Gleeson 2009). However, no study has examined the effect of exercise modality on the s-IgA secretion rate among the same subjects; thus, the exercise intensity and/or duration, which reduces s-IgA secretion, remains unclear. Elucidating the effects of exercise intensity and duration on immune function is important for individuals who exercise to improve their health and physical fitness.

Herein, we hypothesized that a single bout of acute exercise of higher intensity or longer duration would induce a decrease in s-IgA secretion. Therefore, we aimed to clarify the changes in s-IgA secretion in response to acute exercise at different intensities or durations in the same subjects. This study examined the effects of different exercise intensities (Experiment 1) or durations (Experiment 2) by acute exercise on s-IgA secretory responses. Furthermore, the induction of circulating cortisol secretion via sympathetic nervous system activation affects the saliva flow rate during acute exercise (Chicharro et al. 1998). Therefore, in this study, we examined the effects of salivary cortisol concentration, as an index of systemic secretion, on different exercise intensities and/or durations and whether salivary cortisol is associated with the s-IgA secretion response to acute exercise. This study clarified, for the first time, the changes in s-IgA secretion after acute exercise at different exercise intensities or durations.

## Methods

### Subjects

Twelve healthy untrained young males with no exercise habits (mean ± SD: age 21.7 ± 1.6 years; height 174.6 ± 3.6 cm; body weight 65.6 ± 5.2 kg; ${\dot{\text{V}}}$O_2max_ 41.4 ± 4.3 ml/kg/min; maximal heart rate (HRmax) 197.6 ± 5.3 beats/min) participated in this study. In this study, individuals were excluded if, in the last year, they had physiological disorders or chronic diseases, were currently taking any medications, smoked or consumed alcohol daily, or exercised more than 3 h/week. All participants were given verbal and written briefings on the study and provided written informed consent. This study was approved by the Ethics Committee of Ritsumeikan University (BKC-LSMH-2021–054) and conducted in accordance with the Declaration of Helsinki.

### Experimental procedures

**Fig. 1:**
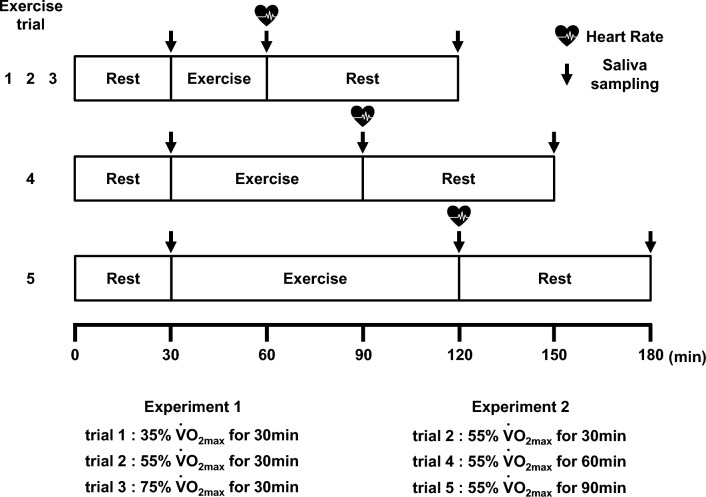
Experimental protocol in this study. Experiment 1: Trial 1 (cycling exercise for 30 min at 35% ${\dot{\text{V}}}$O_2max_), Trial 2 (cycling exercise for 30 min at 55% ${\dot{\text{V}}}$O_2max_), Trial 3 (cycling exercise for 30 min at 75% ${\dot{\text{V}}}$O_2max_). Experiment 2: Trial 2 (cycling exercise for 30 min at 55% ${\dot{\text{V}}}$O_2max_), Trial 4 (cycling exercise for 60 min at 55% ${\dot{\text{V}}}$O_2max_), Trial 5 (cycling exercise for 90 min at 55% ${\dot{\text{V}}}$O_2max_). ${\dot{\text{V}}}$O_2max_: maximal oxygen uptake

${\dot{\text{V}}}$O_2max_ was measured approximately 1 week before the main trials. The cycling exercise load for each exercise trial was calculated from the relationship between ${\dot{\text{V}}}$O_2max_ and work rate, equivalent to 35% ${\dot{\text{V}}}$O_2max_, 55% ${\dot{\text{V}}}$O_2max_, and 75% ${\dot{\text{V}}}$O_2max_. Subjects were instructed not to perform excessive exercise and not to consume both caffeine and alcohol for 24 h before each exercise trial. The subjects performed each exercise trial after fasting and drinking water from 10:00 PM. Hydration status was standardized to 500 ml before each exercise trial (Li and Gleeson 2005). The subjects were instructed to drink 250 ml water before bedtime and after waking up. To eliminate the effect of the circadian rhythm on the saliva flow rate and s-IgA concentration (Hucklebridge et al. 1998; Dimitriou et al. 2002), each measurement was conducted from 9:00 AM to 12:00 PM. The participants arrived at the laboratory at 9:00 AM and remained in a sitting position for 30 min before each exercise trial (Fig. 1). Body weight was measured using a digital platform scale (Innerscan DUAL; TANITA, Tokyo, Japan), and hydration status was checked at rest before exercise; it was confirmed that there was no change between each exercise trial within the subjects (Walsh et al. 2004). This was a randomized crossover study. After each exercise trial, the subjects were allowed to rest in a sitting position for 60 min. The heart rate (HR) (WEP-5204; Nihon Kohden, Tokyo, Japan) was measured 1 min before the end of exercise. Saliva samples were collected before (baseline), immediately after (post), and 60 min after the end of exercise (post-60) according to a previous study (Allgrove et al. 2008). The participants were instructed to fast and not drink anything until 60 min after the end of the exercise. The laboratory temperature and relative humidity were 23.1 ± 1.5 °C and 71.2 ± 12.1%, respectively.

### Exercise trials

All subjects randomly performed five exercise trials (Trial 1: cycling exercise for 30 min at 35% ${\dot{\text{V}}}$O_2max_, Trial 2: cycling exercise for 30 min at 55% ${\dot{\text{V}}}$O_2max_, Trial 3: cycling exercise for 30 min at 75% ${\dot{\text{V}}}$O_2max_, Trial 4: moderate-intensity continuous cycling exercise at 55% ${\dot{\text{V}}}$O_2max_ for 60 min, Trial 5: moderate-intensity continuous cycling exercise at 55% ${\dot{\text{V}}}$O_2max_ for 90 min) with a washout period of at least 3 days between each trial as a reference to a previous study (Allgrove et al. 2008). To determine the effect of exercise intensity on salivary parameters (Experiment 1), continuous cycling exercise at three different exercise intensities (35% [low], 55% [moderate], and 75% ${\dot{\text{V}}}$O_2max_ [high]) was performed for 30 min. Additionally, to determine the effect of exercise duration on salivary parameters (Experiment 2), continuous cycling exercise at 55% ${\dot{\text{V}}}$O_2max_ as a moderate-intensity exercise at three different exercise durations (30 min [short], 60 min [medium], and 90 min [long]) was performed. In this study, considering the burden on the participant, 55% ${\dot{\text{V}}}$O_2max_ for 30 min was performed only once and the duplicate was not implemented in either experiment. Furthermore, it was divided into 30-min intervals, and exercise trials were set for 30 min (short duration), 60 min (medium duration), and 90 min (long duration).

### Measurement of ${\dot{\text{V}}}$O_2max_

${\dot{\text{V}}}$O_2max_ was determined during an incremental cycling exercise test using a cycle ergometer (828E; Monark, Stockholm, Sweden) by monitoring breath-by-breath oxygen consumption and carbon dioxide production (AE-310SRD; Minato, Osaka, Japan). The subjects were instructed to maintain a minimum pedaling rate of 60 rpm according to the protocol of a previous study (Hasegawa et al. 2016). Following a 5-min warm-up at 60 W, they began cycling at 60 W ± 30 W in increments of 15 W each minute until exhaustion (Hasegawa et al. 2016). During the incremental cycling exercise test, the heart rate (HR) (WEP-5204; Nihon Kohden, Tokyo, Japan) and rating of perceived exertion (RPE) were continuously measured every minute during the cycling exercise. The test was considered valid if at least three of the four following criteria were met: [1] plateau in ${\dot{\text{V}}}$O_2_ with an increase in maximal effort, [2] HRmax of the age-predicted maximum (220-age ± 5 beats/min), [3] maximal respiratory exchange ratio of ≧1.1, and [4] an RPE of ≧18 (Borg 1982; American College of Sports Medicine 2000).

### Saliva collection and analysis

Saliva samples were collected between 9:00 AM and 1:00 PM. According to a previous study (Usui et al. 2011), the subjects rinsed their mouths with distilled water (30 s × 3 times). Saliva production was stimulated by chewing a piece of paraffin wax (B.S.A paraffin wax; B.S.A, Aichi, Japan) for 1 min at a frequency of 1 chew/sec (Libicz et al. 2006; Balsalobre-Fernández et al. 2014; Gomar-Vercher et al. 2018). The collected saliva was separated from the paraffin wax by centrifugation at 4 °C, 1500 g, for 5 min. The saliva flow rate (ml/min) was measured by weighing and the saliva density was estimated to be 1.0 g/ml (Usui et al. 2011). Saliva supernatants were stored at − 20 °C until analysis.

Salivary IgA and cortisol concentrations were measured using an enzyme-linked immunosorbent assay (Salimetrics, State College, PA, USA) according to previous studies (Mc Naughton et al. 2006; Peñailillo et al. 2015). The absorbance was measured at 450 nm using a microplate reader and an xMark microplate spectrophotometer (Bio-Rad Laboratories, Hercules, CA, USA). The salivary IgA secretion rate (μg/min) was calculated from the saliva flow rate per minute (ml/min) and salivary IgA concentration (μg/ml), according to a previous study (Koch et al. 2007). In this study, salivary cortisol concentrations were measured based on the assumption that the concentration of cortisol in the saliva reflects the concentration secreted into the blood (Chicharro et al. 1998; Hackney and Walz 2013). The average coefficients of variation for IgA and cortisol assays were 3.3% and 2.6%, respectively.

### Statistical analysis

All statistical analyses were performed using the StatView software (5.0; SAS Institute, Tokyo, Japan). All values are expressed as mean ± SD. In the Experiment 1, the saliva flow rate, s-IgA concentration, salivary cortisol concentration, and s-IgA secretion rate at different exercise intensities (35%, 55%, and 75% ${\dot{\text{V}}}$O_2max_) were compared using three trials × three time points (baseline, post, and post-60) of a two-way repeated-measures ANOVA. Fisher’s post-hoc test was used to correct for multiple comparisons when the ANOVA revealed significant differences. The relationship between the change in saliva flow rate and the change in salivary cortisol concentration was determined using Pearson’s correlation coefficient. In the Experiment 2, the comparison of saliva flow rate, s-IgA concentration, salivary cortisol concentration, and s-IgA secretion rate at different exercise durations (30, 60, and 90 min) was performed using three trials × three time points (baseline, post, and post-60) in a two-way repeated-measures ANOVA. Fisher’s post-hoc test was used to correct for multiple comparisons when the ANOVA revealed significant differences. Statistical significance was defined as P < 0.05. We calculated the effect size of 0.25 (large) for the two-way repeated-measures ANOVA using G*Power (version 3.1), which determined the sample size needed for this study. To detect the effect size at alpha levels of 0.05 and 80% power, the sample size was set to 12 participants.

## Results

The HRs recorded 1 min before the end of each exercise trial were as follows:

Experiment 1: 35% ${\dot{\text{V}}}$O_2max_; 55% ${\dot{\text{V}}}$O_2max_; 75% ${\dot{\text{V}}}$O_2max_ (112.6 ± 13.9; 150.8 ± 10.6; 186.4 ± 7.7 beats/min, respectively, data not shown).

Experiment 2: 30 min; 60 min; 90 min (150.8 ± 10.6; 158.7 ± 13.4; 165.6 ± 12.6 beats/min, respectively, data not shown).

Experiment 1: exercise intensity study.

No significant differences in saliva flow rate, s-IgA concentration, or s-IgA secretion rate were observed among the three exercise intensity trials (Table 1). However, there was a significant interaction of salivary cortisol concentrations between the three trials (35%, 55%, and 75% ${\dot{\text{V}}}$O_2max_) and three time points (baseline, post, and post-60) (F = 4.470, interaction: P < 0.01, Table 1). The salivary cortisol concentration in the 75% ${\dot{\text{V}}}$O_2max_ trial at post and post-60 were significantly higher than that at baseline (P < 0.05 respectively; Table 1). The salivary cortisol concentration in the 75% ${\dot{\text{V}}}$O_2max_ trial was significantly higher than that in the 35% ${\dot{\text{V}}}$O_2max_ trial at post and post-60 (P < 0.01, respectively; Table 1). The 75% ${\dot{\text{V}}}$O_2max_ trial was significantly higher than the 55% ${\dot{\text{V}}}$O_2max_ trial at post and post-60 (P < 0.01, respectively; Table 1).

**Table 1:** Effects of different exercise intensities on the saliva parameters

	Trial	Baseline	Post	Post-60	Two-way ANOVA	
					F-value	P-value
Saliva flow rate (ml/min)	35% ${\dot{\text{V}}}$O_2max_	2.42 ± 0.91	2.14 ± 0.96	2.64 ± 1.03	Trial: 1.076	Trial: 0.345
	55% ${\dot{\text{V}}}$O_2max_	2.13 ± 0.77	1.90 ± 0.99	2.41 ± 1.17	Time: 4.841	Time: 0.001
	75% ${\dot{\text{V}}}$O_2max_	2.35 ± 0.79	1.50 ± 0.88	2.48 ± 0.70	Interaction: 0.486	Interaction: 0.746
Salivary IgA concentration (μg/ml)	35% ${\dot{\text{V}}}$O_2max_	92.79 ± 70.17	110.61 ± 86.92	85.53 ± 69.51	Trial: 0.531	Trial: 0.590
	55% ${\dot{\text{V}}}$O_2max_	102.09 ± 75.65	139.43 ± 108.37	86.14 ± 68.01	Time: 1.826	Time: 0.167
	75% ${\dot{\text{V}}}$O_2max_	111.34 ± 99.18	142.25 ± 143.18	101.39 ± 70.12	Interaction: 0.077	Interaction: 0.989
Salivary IgA secretion rate (μg/min)	35% ${\dot{\text{V}}}$O_2max_	201.41 ± 132.82	198.79 ± 126.32	194.59 ± 138.93	Trial: 0.143	Trial: 0.867
	55% ${\dot{\text{V}}}$O_2max_	185.70 ± 92.54	205.00 ± 82.63	161.33 ± 84.69	Time: 0.271	Time: 0.763
	75% ${\dot{\text{V}}}$O_2max_	217.18 ± 138.89	141.17 ± 81.09	220.14 ± 124.63	Interaction: 1.017	Interaction: 0.402
Salivary cortisol concentration (μg/dl)	35% ${\dot{\text{V}}}$O_2max_	0.13 ± 0.04	0.13 ± 0.05	0.09 ± 0.04^§¶^	Trial: 21.167	Trial: 0.001
	55% ${\dot{\text{V}}}$O_2max_	0.19 ± 0.07^*^	0.18 ± 0.07	0.11 ± 0.07^§¶^	Time: 1.616	Time: 0.204
	75% ${\dot{\text{V}}}$O_2max_	0.18 ± 0.06	0.35 ± 0.13^†‡#^	0.35 ± 0.30^†‡#^	Interaction: 4.470	Interaction: 0.002

Values are means ± SD. ${\dot{\text{V}}}$O_2max_: maximal oxygen uptake. IgA: Immunoglobulin A

*:P < 0.05, vs. baseline at 35%${\dot{\text{V}}}$O_2max_. †:P < 0.01, vs. same time point at 35% ${\dot{\text{V}}}$O_2max_. ‡:P < 0.01, vs. same time point at 55% ${\dot{\text{V}}}$O_2max_. §:P < 0.01, vs. baseline in each trial. ¶:P < 0.05, vs. post in each trial. #:P < 0.05, vs. baseline at 75%${\dot{\text{V}}}$O_2max_

**Fig. 2:**
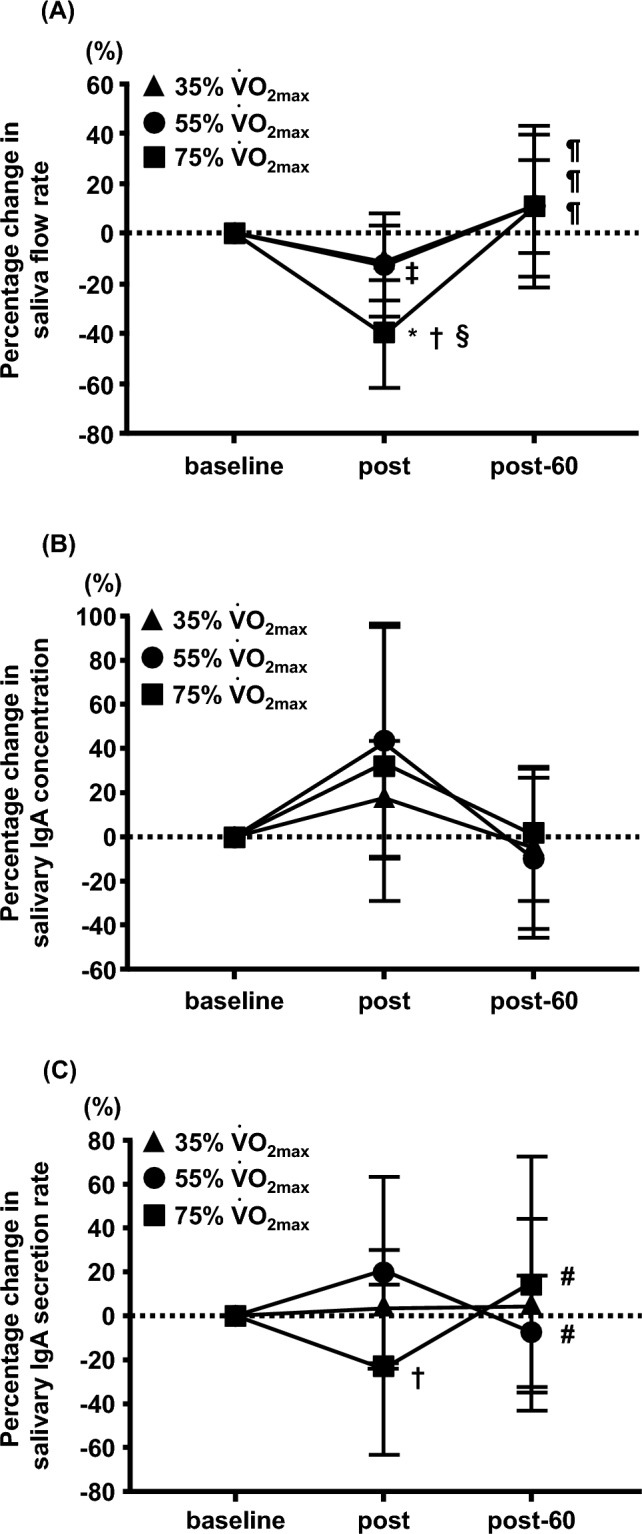
Effects of different exercise intensities on the percentage change in saliva flow rate (**A**), salivary IgA concentration (**B**), and salivary IgA secretion rate (**C**) from baseline in healthy untrained young males (n = 12). Data are expressed as mean ± SD. IgA: Immunoglobulin A; ${\dot{\text{V}}}$O_2max_: maximal oxygen uptake. *P < 0.01 vs post at 35% ${\dot{\text{V}}}$O_2max_. †P < 0.01 vs post at 55% ${\dot{\text{V}}}$O_2max_. ‡P < 0.05 vs baseline at 35% ${\dot{\text{V}}}$O_2max_. §P < 0.01 vs baseline at 75% ${\dot{\text{V}}}$O_2max_. ¶P < 0.01 vs post in each trial. #P < 0.05 vs post in each trial

There was a significant interaction in the percentage change in the saliva flow rate between the three trials (35%, 55%, and 75% ${\dot{\text{V}}}$O_2max_) and the three time points (baseline, post, and post-60) (F = 2.815, interaction: P < 0.05, Fig. 2A). The percentage change in saliva flow rate in the 35% ${\dot{\text{V}}}$O_2max_ and 75% ${\dot{\text{V}}}$O_2max_ trials at post was significantly lower than that at baseline (35% ${\dot{\text{V}}}$O_2max_ trial, P < 0.05; 75% ${\dot{\text{V}}}$O_2max_ trial, P < 0.01; Fig. 2A). In addition, the percentage change in saliva flow rate in the 75% ${\dot{\text{V}}}$O_2max_ trial was significantly lower than that in the 35% ${\dot{\text{V}}}$O_2max_ and 55% ${\dot{\text{V}}}$O_2max_ trials at post (P < 0.01, Fig. 2A). In contrast, the percentage change in saliva flow rate in the 35% ${\dot{\text{V}}}$O_2max_ and 75% ${\dot{\text{V}}}$O_2max_ trials post-60 was significantly higher than that at post (35% ${\dot{\text{V}}}$O_2max_ trial, P < 0.01; 75% ${\dot{\text{V}}}$O_2max_ trial, P < 0.01; Fig. 2A). The percentage changes in s-IgA concentrations were not significantly different between the three exercise intensity trials (Fig. 2B). There was a significant interaction between the percentage change in the s-IgA secretion rate between the three trials (35%, 55%, and 75% ${\dot{\text{V}}}$O_2max_) and the three time points (baseline, post, and post-60) (F = 3.365, P < 0.05, Fig. 2C). The percentage change in the s-IgA secretion rate in the 75% ${\dot{\text{V}}}$O_2max_ trial was significantly lower than that in the 55% ${\dot{\text{V}}}$O_2max_ trial (P < 0.01, Fig. 2C). The 75% ${\dot{\text{V}}}$O_2max_ trial post-60 was significantly higher than that at post (P < 0.05, Fig. 2C).

**Fig. 3:**
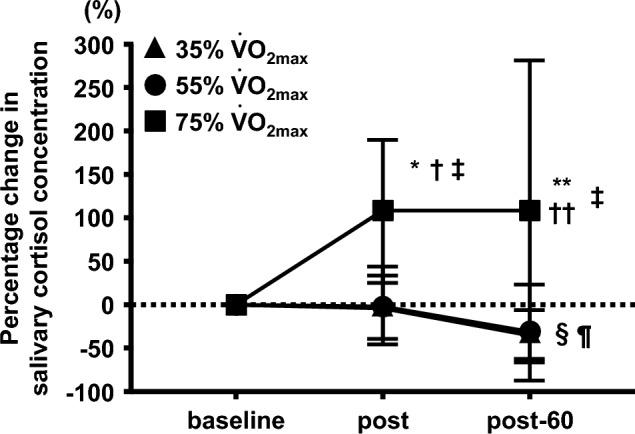
Effects of different exercise intensities on the percentage change in salivary cortisol concentration from baseline in healthy untrained young males (n = 12). Data are expressed as mean ± SD. ${\dot{\text{V}}}$O_2max_: maximal oxygen uptake. *P < 0.01 vs post at 35% ${\dot{\text{V}}}$O_2max_. **P < 0.01 vs post-60 at 35% ${\dot{\text{V}}}$O_2max_. †P < 0.01 vs post at 55% ${\dot{\text{V}}}$O_2max_. ††P < 0.01 vs post-60 at 55% ${\dot{\text{V}}}$O_2max_. ‡P < 0.05 vs baseline at 75% ${\dot{\text{V}}}$O_2max_. §P < 0.01 vs baseline at 35% ${\dot{\text{V}}}$O_2max_. ¶P < 0.01 vs post at 35% ${\dot{\text{V}}}$O_2max_

There was a significant interaction of the percentage change in salivary cortisol concentration between the three trials (35%, 55%, and 75% ${\dot{\text{V}}}$O_2max_) and the three time points (baseline, post, and post-60) (F = 4.469, interaction: P < 0.05, Fig. 3). The percentage changes in salivary cortisol concentration at post and post-60 were significantly higher than that at baseline in the 75% ${\dot{\text{V}}}$O_2max_ trial (P < 0.05, respectively; Fig. 3). The percentage change in salivary cortisol concentration in the 75% ${\dot{\text{V}}}$O_2max_ trial was significantly higher than that in the 35% ${\dot{\text{V}}}$O_2max_ trial at post and post-60 (P < 0.01, respectively; Fig. 3). The percentage change in salivary cortisol concentration in the 75% ${\dot{\text{V}}}$O_2max_ trial was significantly higher than that in the 55% ${\dot{\text{V}}}$O_2max_ trial at post and post-60 (P < 0.01, respectively; Fig. 3).

**Fig. 4:**
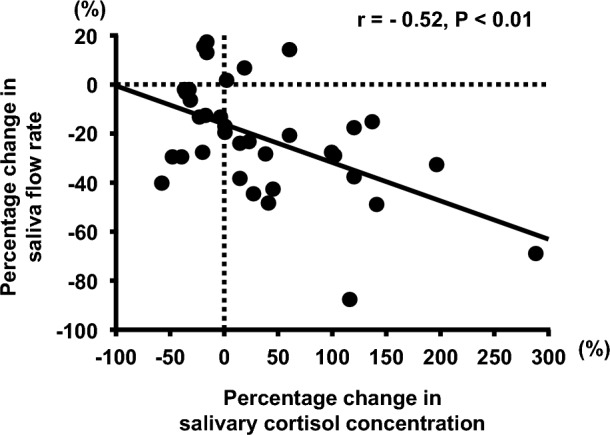
Correlation between percentage changes in salivary cortisol concentration and percentage changes in saliva flow rate among different exercise intensities in healthy untrained young males (n = 12)

A significant negative correlation of percentage changes between saliva flow rate and salivary cortisol concentration was observed from baseline to post in each exercise trial (r = -0.52, P < 0.01, Fig. 4).

Experiment 2: exercise duration study.

**Fig. 5:**
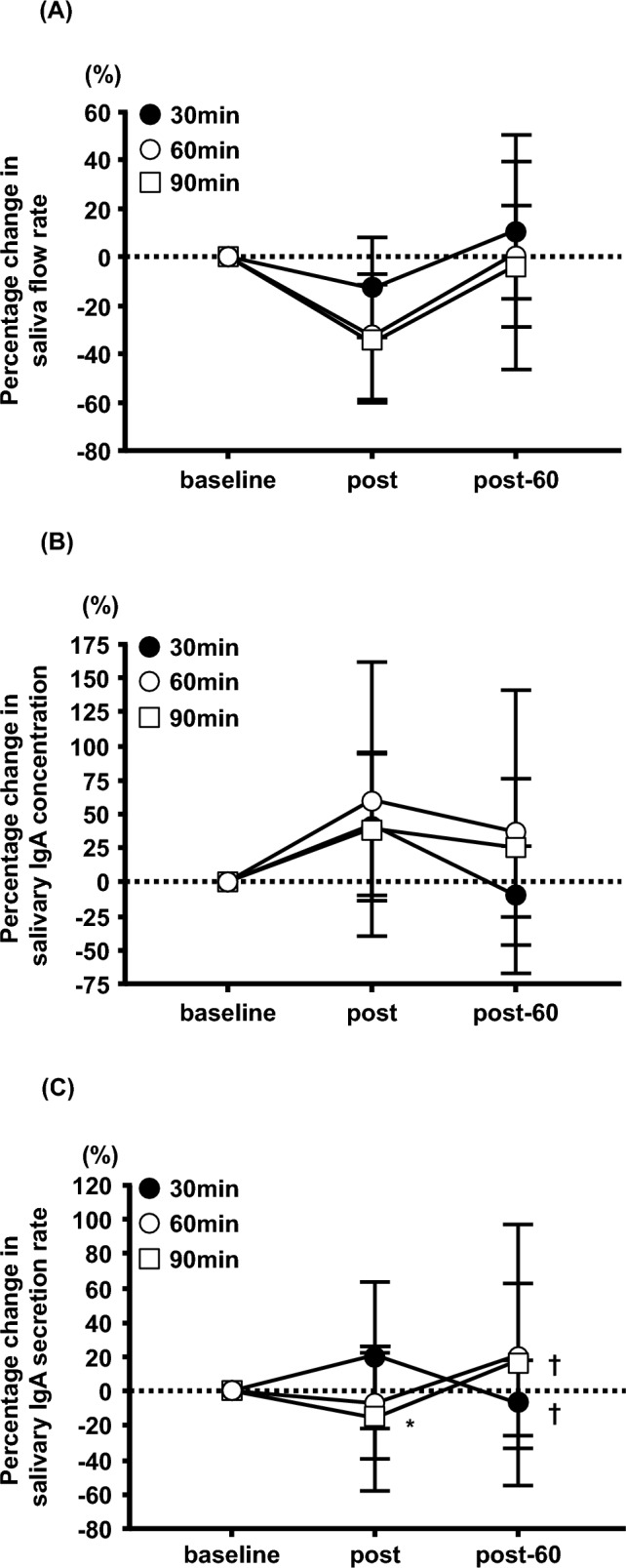
Effects of different exercise durations on the percentage change in saliva flow rate (**A**), salivary IgA concentration (**B**), and salivary IgA secretion rate (**C**) from baseline in healthy untrained young males (n = 12). Data are expressed as mean ± SD. IgA: Immunoglobulin A. *P < 0.05 vs post at 30 min. †P < 0.05 vs post in each trial

No significant differences were observed in saliva flow rate, s-IgA concentration, s-IgA secretion rate, or salivary cortisol concentration in each exercise trial (Table 2). There was no significant interaction between the percentage change in saliva flow rate and s-IgA concentration (Fig. 5A, B). However, the percentage change in the s-IgA secretion rate showed a significant interaction between the three trials (30, 60, and 90 min) and the three time points (baseline, post, and post-60) (F = 2.471, P < 0.05, Fig. 5C). The percentage change in the s-IgA secretion rate in the 90-min trial was significantly lower than that in the 30-min trial at post (P < 0.05, Fig. 5C). The percentage change in the s-IgA secretion rate in the 90-min trial at post-60 was significantly higher than that at post (P < 0.05, Fig. 5C).

**Table 2:** Effects of different exercise durations on the saliva parameters

	Trial	Baseline	Post	Post-60	Two-way ANOVA	
					F-value	P-value
Saliva flow rate (ml/min)	30 min	2.13 ± 0.77	1.90 ± 0.99	2.41 ± 1.17	Trial: 1.114	Trial: 0.332
	60 min	2.63 ± 0.90	1.79 ± 0.94	2.52 ± 0.92	Time: 4.327	Time: 0.016
	90 min	2.28 ± 1.11	1.58 ± 0.99	2.07 ± 0.88	Interaction: 0.408	Interaction: 0.802
Salivary IgA concentration (μg/ml)	30 min	102.09 ± 75.65	139.43 ± 108.37	86.14 ± 68.01	Trial: 1.003	Trial: 0.371
	60 min	100.07 ± 67.08	156.11 ± 131.14	109.91 ± 77.01	Time: 1.875	Time: 0.159
	90 min	115.67 ± 83.24	162.79 ± 151.04	155.21 ± 154.79	Interaction: 0.255	Interaction: 0.906
Salivary IgA secretion rate (μg/min)	30 min	185.70 ± 92.54	205.00 ± 82.63	161.33 ± 84.69	Trial: 1.199	Trial: 0.306
	60 min	223.84 ± 104.04	198.23 ± 92.26	249.66 ± 150.27	Time: 0.791	Time: 0.456
	90 min	199.35 ± 64.81	160.78 ± 81.03	250.19 ± 179.97	Interaction: 1.185	Interaction: 0.322
Salivary cortisol concentration (μg/dl)	30 min	0.19 ± 0.07	0.18 ± 0.07	0.11 ± 0.07	Trial: 5.653	Trial: 0.005
	60 min	0.19 ± 0.12	0.22 ± 0.11	0.14 ± 0.07	Time: 3.784	Time: 0.026
	90 min	0.21 ± 0.09	0.31 ± 0.20	0.23 ± 0.17	Interaction: 0.874	Interaction: 0.483

Values are means ± SD

IgA: Immunoglobulin A

**Fig. 6:**
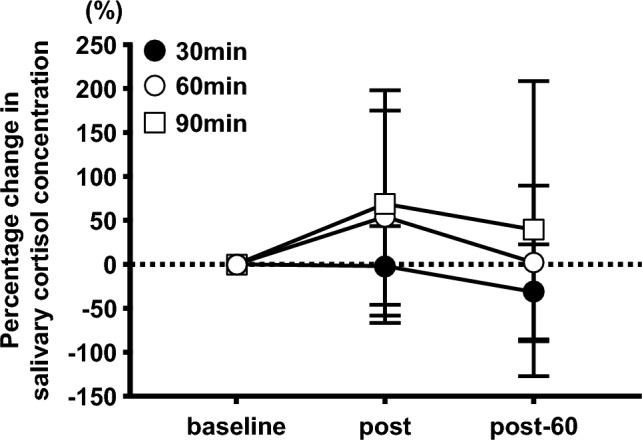
Effects of different exercise durations on the percentage change in salivary cortisol concentration from baseline in healthy untrained young males (n = 12). Data are expressed as mean ± SD

There was no significant interaction effect in the percentage change in salivary cortisol concentrations (Fig. 6).

## Discussion

The main findings of the experiment 1 revealed that the s-IgA secretion rate was transiently reduced after cycling exercise at 75% ${\dot{\text{V}}}$O_2max_ in healthy untrained young men, as examined using 30-min acute exercise at different intensities (35%, 55%, and 75% ${\dot{\text{V}}}$O_2max_). Furthermore, the experiment 2 showed a reduction in the s-IgA secretion rate after cycling exercise for 90 min, as examined using an acute 55% ${\dot{\text{V}}}$O_2max_ bout of exercise at different exercise durations (30, 60, and 90 min). Since s-IgA neutralizes the invasion of viruses and bacteria into the oral cavity, it is important to maintain oral s-IgA secretions (Lamm et al.^1995^; Marcotte and Lavoie 1998; Woof and Mestecky 2005). These results suggest that depression in s-IgA secretion is transiently induced by exercise intensity of greater than or equal to 75% ${\dot{\text{V}}}$O_2max_ for 30 min or exercise duration of greater than or equal to 90 min at 55% ${\dot{\text{V}}}$O_2max_ exercise intensity in healthy untrained young men.

Previous studies examining the response of s-IgA secretion to transient cycling exercise have shown no consistent evidence that the rate of s-IgA secretion is reduced or unchanged by various exercise intensities (Allgrove et al. 2008; Matsubara et al. 2010; Usui et al. 2011; Murase et al. 2016) or durations (Mackinnon and Hooper 1994; Laing et al. 2005; Allgrove et al. 2008). MacDowell et al. (1991) examined the effects of different exercise intensities (50, 65, and 80% ${\dot{\text{V}}}$O_2max_) with the same exercise duration (20 min) or different durations (15, 30, and 45 min) with the same exercise intensity (60% ${\dot{\text{V}}}$O_2max_) on s-IgA concentration but did not examine the same subjects to compare exercise intensity and duration. Moreover, they only assayed IgA concentration (McDowell et al. 1991). However, measurement of the IgA secretion rate by multiplying the s-IgA concentration and saliva flow rate may be more accurate because the s-IgA concentration is decreased/increased by the saliva flow rate (Bishop and Gleeson 2009). To clarify these issues, the present study compared different exercise intensities and durations in the same subjects. Consequently, the experiment 1 showed no change in the s-IgA secretion rate after cycling exercise at 35% and 55% ${\dot{\text{V}}}$O_2max_ exercise intensities for 30 min, whereas the change in the 75% ${\dot{\text{V}}}$O_2max_ trial was significantly lower than that in the 55% ${\dot{\text{V}}}$O_2max_ trial immediately after exercise (-45.7%). Additionally, the s-IgA secretion rate after cycling exercise at 55% ${\dot{\text{V}}}$O_2max_ exercise intensity for 30 and 60 min was not statistically significant; however, a − 37.0% decrease in the s-IgA secretion rate after cycling exercise for 90 min was observed compared to the 30-min trial in the experiment 2. Furthermore, in this study, the secretion rate during exercise for 30–90 min was dependent on time, and this at 35–75% ${\dot{\text{V}}}$O_2max_ exercise intensities was dependent on exercise intensity. Mackinnon et al. (1993) showed that the s-IgA secretion rate decreased by 27% after a 30-min interval exercise (Mackinnon et al. 1993). In addition, Laing et al. (2005) and Nieman et al. (2003) showed that long-term exercise (120 min and 13 h) decreased s-IgA secretion rates by 34% and 50%, respectively (Nieman et al. 2003; Laing et al. 2005). Therefore, in the case of much higher intensities or longer-term exercise than those in the present study, the s-IgA secretion rate may decrease even more.

In the experiment 1, the saliva flow rate and s-IgA secretion rate transiently decreased after cycling exercise at 75% ${\dot{\text{V}}}$O_2max_ for 30 min. Saliva secretion is regulated by the autonomic nervous system (Chicharro et al. 1998). Therefore, after cycling exercise using an exercise intensity that exceeds the anaerobic threshold, such as 90% HRmax and increased exercise intensity, the saliva flow rate decreases through increased sympathetic nervous activity (Chicharro et al. 1998; Jamnick et al. 2020). In the experiment 1, the HR during each cycling exercise was equivalent to 57.0% HRmax for 35% ${\dot{\text{V}}}$O_2max_ exercise intensity_,_ 76.3% HRmax for 55% ${\dot{\text{V}}}$O_2max_, and 94.3% HRmax for 75% ${\dot{\text{V}}}$O_2max_. Therefore, an exercise intensity of 75% ${\dot{\text{V}}}$O_2max_ for 30 min in this study may have decreased the saliva flow rate by increasing sympathetic nervous activity. Several studies have shown that exercise-induced hypothalamic–pituitary–adrenal (HPA) axis activity may regulate the saliva flow rate via adrenergic stimulation by enhancing circulating cortisol secretion from the adrenal cortex (Chicharro et al. 1998; Hackney and Walz 2013). In this experiment 1, an increase in salivary cortisol concentration, an index of systemic secretion, was observed after cycling exercise at 75% ${\dot{\text{V}}}$O_2max_ for 30 min, but it did not change after cycling exercise at 35% and 55% ${\dot{\text{V}}}$O_2max_. Furthermore, a significant negative correlation was found between the change in saliva flow rate and change in salivary cortisol concentration before and after exercise (r = − 0.52, P < 0.01), suggesting that the change in cortisol secretion may be involved in the decrease in saliva flow rate. Therefore, high-intensity exercise-induced upregulation of the autonomic nervous system and/or HPA axis activity may have caused the decrease in the s-IgA secretion rate by decreasing the saliva flow rate at 75% ${\dot{\text{V}}}$O_2max_ cycling exercise for 30 min.

In this study, the s-IgA secretion rate was transiently reduced after cycling exercise at 55% ${\dot{\text{V}}}$O_2max_ for 90 min; however, no significant changes in the saliva flow rate or s-IgA concentration were observed. Walsh et al. (2004) have shown decreased saliva flow rates after cycling exercise at 60% ${\dot{\text{V}}}$O_2max_ for 100 min (Walsh et al. 2004). Furthermore, Mc Naughton et al. (2006) have showed reduced s-IgA concentrations after cycling exercise at 60% ${\dot{\text{V}}}$O_2max_ for 90 min (Mc Naughton et al. 2006). As the s-IgA secretion rate was calculated by multiplying the s-IgA concentration and saliva flow rate, a slight decrease in s-IgA concentration and saliva flow rate, which were not statistically visible, may have led to the reduction of the s-IgA secretion rate in this study. To test this hypothesis, it is necessary to examine using the protocol of exercise durations for more than 90 min.

This study had certain limitations. First, in the experiment 1, s-IgA secretion decreased compared to that before exercise in 5 out of 12 subjects at 35% ${\dot{\text{V}}}$O_2max_, 3 at 55% ${\dot{\text{V}}}$O_2max_, and 9 at 75% ${\dot{\text{V}}}$O_2max_. Furthermore, in the experiment 2, s-IgA secretion decreased compared to that before exercise in 3 out of 12 subjects at 30 min, 7 at 60 min, and 8 at 90 min. Thus, the threshold of exercise intensity or duration required to reduce s-IgA secretion may vary depending on the individuals. The threshold of intensity or duration required for exercise to induce the same decrease in s-IgA secretion may be higher or longer, respectively. Further studies are required to examine the response of s-IgA to higher exercise intensities or longer exercise durations. Second, the experiments 1 and 2 did not measure fluid intake during or after exercise. In the experiment 1, only four participants had measured their weight preliminarily before and after exercise: 35% ${\dot{\text{V}}}$O_2max_ exercise intensity for 30-min had a 0.2% decrease, 55% ${\dot{\text{V}}}$O_2max_ had a 0.4% decrease, and 75% ${\dot{\text{V}}}$O_2max_ had a 0.7% decrease after exercise. In the experiment 2, 55% ${\dot{\text{V}}}$O_2max_ exercise intensity for 30-min had a 0.4% decrease, 60-min had a 0.8% decrease, and 90-min had a 1.3% decrease after exercise. Previous studies have reported that a decrease in saliva flow rate due to dehydration is affected by a decrease in body weight of 3.0% or more (Walsh et al. 2004). However, since this study showed both a trend of increasing dehydration and decreasing saliva flow rate at dehydration levels of less than 3.0% change in body weight, future studies are needed to examine the effects of lower levels of exercise induced dehydration on the saliva flow rate. In addition, these saliva flow rate-related differences might affect the s-IgA and cortisol secretion responses to exercise. Further studies would thus be required to examine the influence of minimized changes in the saliva flow rate.

In conclusion, the present findings suggest a transient decrease in the s-IgA secretion rate in response to acute exercise at greater than or equal to 75% ${\dot{\text{V}}}$O_2max_ exercise intensity for 30 min or at 55% ${\dot{\text{V}}}$O_2max_ exercise intensity for greater than or equal to 90 min in healthy untrained young men.

### Supplementary Information

Below is the link to the electronic supplementary material.

Supplementary file1 (PPTX 91 KB)

## Data Availability

The data that support the findings of this study are available from the corresponding author upon reasonable request.

## Abbreviations

HPA: Hypothalamic–pituitary–adrenal
HR: Heart rate
HRmax: Maximal heart rate
RPE: Rating of perceived exertion
${\dot{\text{V}}}$O_2max_: Maximal oxygen uptake
s-IgA: Salivary immunoglobulin A
