# Supplementary material for: Effects of different exercise intensities or durations on salivary IgA secretion
Source: Eur J Appl Physiol. 2024 Apr 18;124(9):2687–96. doi: 10.1007/s00421-024-05467-6 (PMC11365859; doi:10.1007/s00421-024-05467-6)
Supplement: Supplementary file 1 — Supplementary file1 (PPTX 91 KB) [file 421_2024_5467_MOESM1_ESM.pptx]

## Slide 1
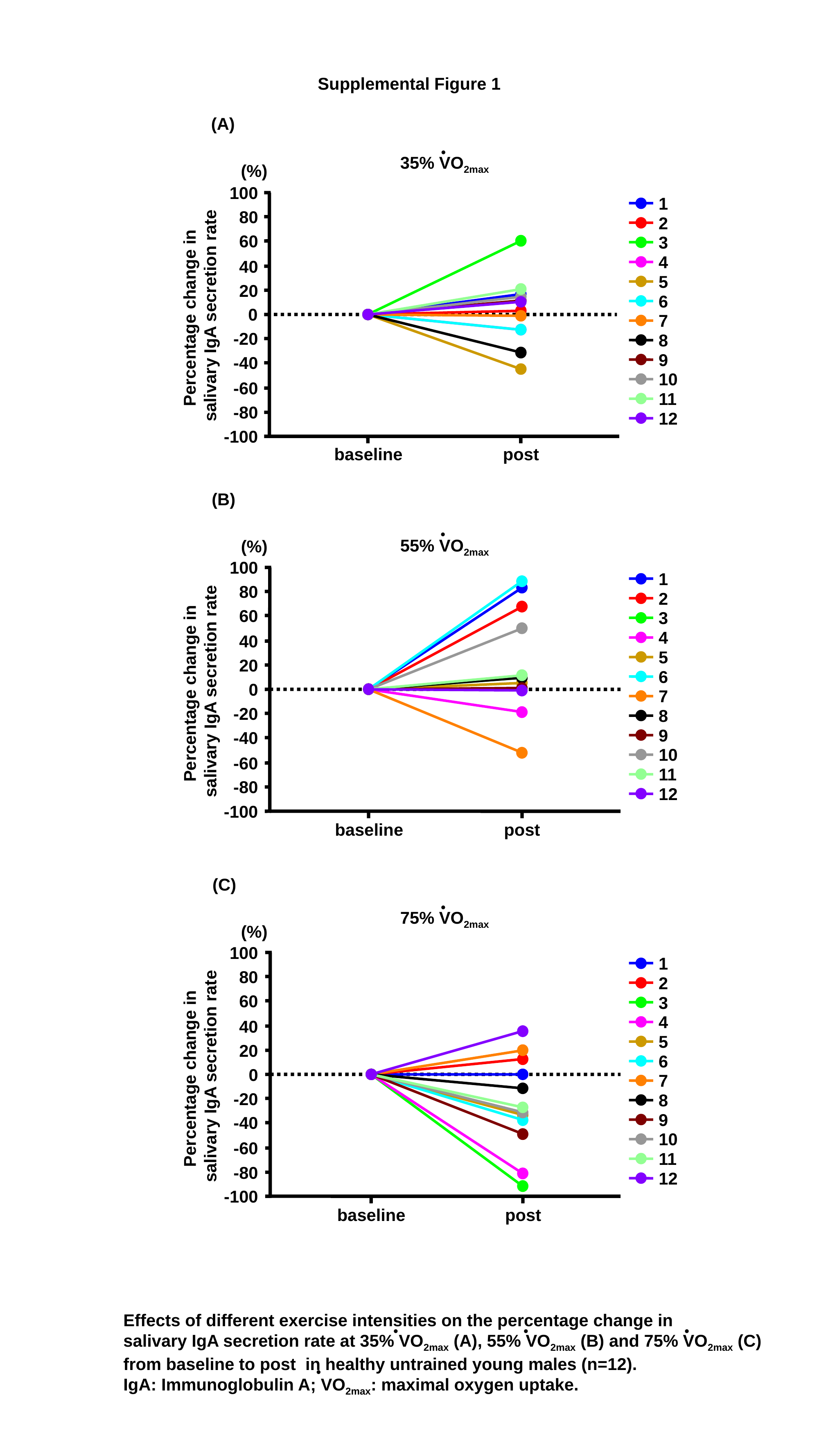

Supplemental Figure 1
(A)
・
35% VO2max
(%)
100
80
60
40
20
0
-20
-40
-60
-80
-100
1
2
3
4
5
6
7
8
9
10
11
12
Percentage change in
salivary IgA secretion rate
baseline
post
(B)
・
55% VO2max
(%)
100
80
60
40
20
0
-20
-40
-60
-80
-100
1
2
3
4
5
6
7
8
9
10
11
12
Percentage change in
salivary IgA secretion rate
baseline
post
(C)
・
75% VO2max
(%)
100
80
60
40
20
0
-20
-40
-60
-80
-100
Percentage change in
salivary IgA secretion rate
baseline
post
1
2
3
4
5
6
7
8
9
10
11
12
Effects of different exercise intensities on the percentage change in
salivary IgA secretion rate at 35% VO2max (A), 55% VO2max (B) and 75% VO2max (C)
from baseline to post in healthy untrained young males (n=12).
IgA: Immunoglobulin A; VO2max: maximal oxygen uptake.
・
・
・
・

## Slide 2
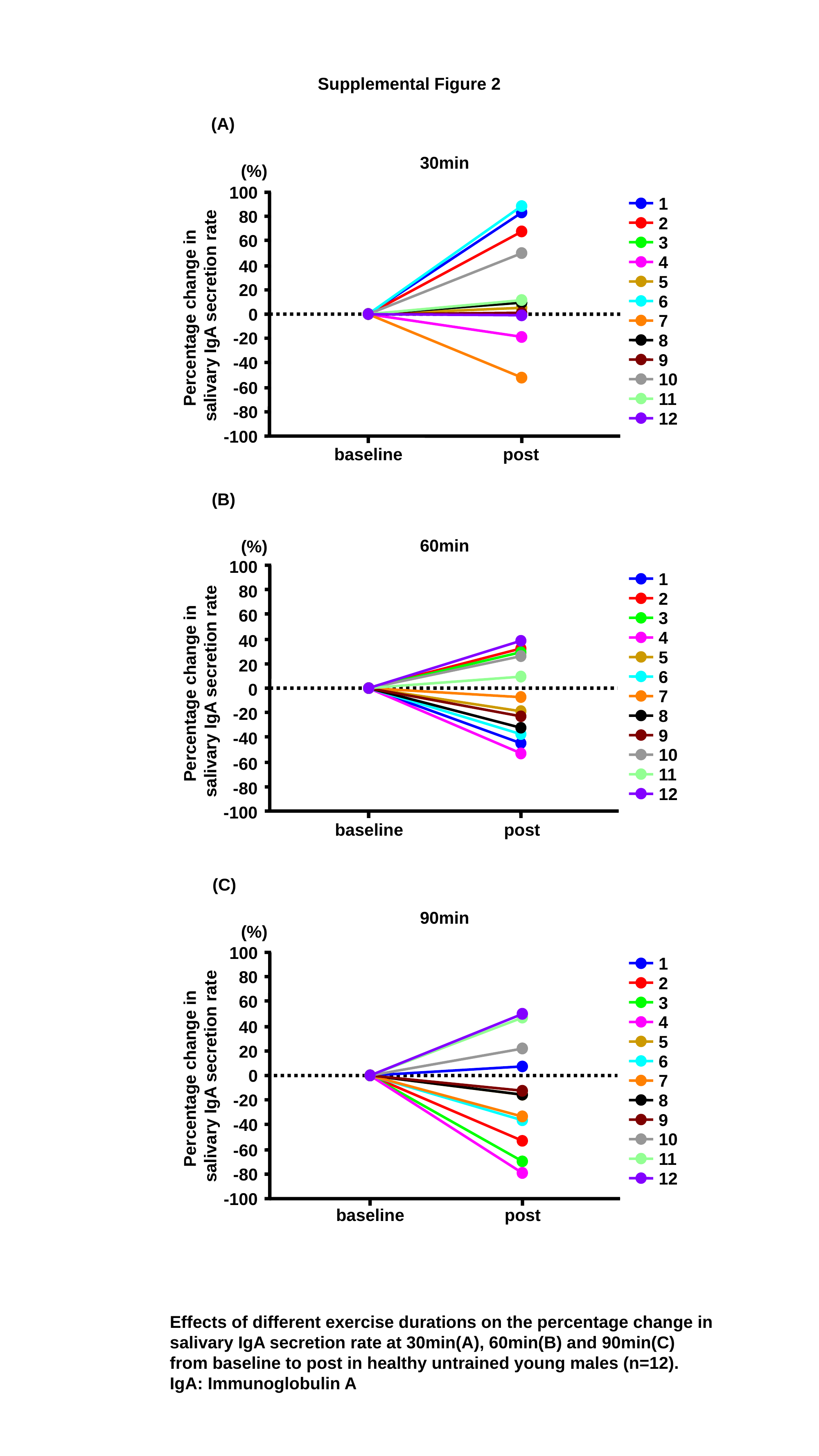

Supplemental Figure 2
(A)
30min
(%)
100
80
60
40
20
0
-20
-40
-60
-80
-100
1
2
3
4
5
6
7
8
9
10
11
12
Percentage change in
salivary IgA secretion rate
baseline
post
(B)
60min
(%)
100
80
60
40
20
0
-20
-40
-60
-80
-100
1
2
3
4
5
6
7
8
9
10
11
12
Percentage change in
salivary IgA secretion rate
baseline
post
(C)
90min
(%)
100
80
60
40
20
0
-20
-40
-60
-80
-100
1
2
3
4
5
6
7
8
9
10
11
12
Percentage change in
salivary IgA secretion rate
baseline
post
Effects of different exercise durations on the percentage change in
salivary IgA secretion rate at 30min(A), 60min(B) and 90min(C)
from baseline to post in healthy untrained young males (n=12).
IgA: Immunoglobulin A
